# Annotations

**Published:** 1910-03-19

**Authors:** 


### Annotations.

### The Late Dr. C. E. Fish.

The death, on March 7 last, of Dr. C. E. Fish, following hard upon that of Dr. Morton, of Nordrach-in-Wales, might seem to reflect upon the ultimate results of sanatorium treatment. This, however, is not wholly the case. After a successful career at Cambridge, Dr. Fish developed symptoms of phthisis when holding a resident appointment at a London hospital. Travel and residence in California proved unavailing, and he only made improvement after a stay, rather late in the day, at Nordrach-im-Schwarzwald, under Dr. Walther. In 1901, with Dr. Crace-Calvert, he started the Vale of Clwyd Sanatorium. Of a reserved though courteous demeanour, Dr. Fish won the respect of his friends by his disinterestedness, his loyalty to conviction, and his refined and happy married life. Up to the last he showed keenness for professional topics, as evidenced by very recent letters to a medical contemporary.

### The Growth of Cremation.

It will be remembered that the late Mr. W. P. Frith, R.A., in his will, laid it down as the duty of the individual to his kind to provide for such final disposal of his body as shall be least detrimental to those who survive him. Incineration, in the artist's view, provided the quickest and safest mode of such disposal, and he therefore left directions for his body to be cremated at his death. This view is shared by an increasing number of people of position and influence, and during 1909 George Meredith, Archdeacon Tribe, Canon Cooper, Thomas Wakley, of the *Lancet*, and Felix Cobbold, M.P. for Ipswich, were cremated at Woking or Golder's Green. In Great Britain the numbers cremated in 1909 were 855, an increase of nearly 8 per cent. over those in 1908, whilst the numbers cremated at Woking and Golder's Green were 526, against 483 in 1908. If all thinking people with any pretension to scientific knowledge or apprehension could be induced to visit, study, and understand the great cemeteries and the system pursued in them in connection with our large urban population, cremation would be so common that the crematoria at present available would have to be increased ten-fold within the year.

### Child Tramps.

One of the admirable points of the Children's Act is the 118th Section, which applies to children who are habitually debarred from attending school owing to the nomadic proclivities of their parents. The first prosecution under this section was only quite recently, when a tramping couple were charged at Sudbury, and their three children, the youngest over the age of five years, were ordered to be sent to an industrial home. The prosecution was undertaken at the instance of the Society for the Prevention of Cruelty to Children, the inspectors of which have been in touch with these poor child tramps for the last three years. This, we know, is no isolated case. There are dozens of tramps wandering about the country, dragging their progeny in their wake, and morally and physically ruining the lives of their children by their peripatetic customs. Such children, in the majority of cases, are underfed, half-starving, and, contrary to what one would expect, seeing that they get all the advantages of a "healthy, hardy open-air existence," as Alderman Cute would have observed, are miserable specimens of boy and girlhood. Their stature is usually much below the average that obtains among rural children, and the percentage of phthisis among tramping children is notably high, even if we overlook the many cases of scrofulous tendencies" which some authorities refuse to class under the heading "tuberculosis." To a large extent their physical deterioration is due to the hardships they have undergone. An open-air life is doubtless very desirable for a growing child, but the Spartan severity which the tramp's child has to endure kills more often than it covers good health, while the moral dangers of such a pariah existence as in the nature of things must be the lot of these children need no comment. Under the Children's Act the authorities have power to step in and rescue the child-tramp by sending him to an industrial school until he has attained his sixteenth year. This is an admirable provision, and it is sincerely to be hoped that the local authorities will avail themselves more frequently in the future of this power which the legislature has given them to deal with an evil which appears to be otherwise untouchable.

### Professor Callendar's Thermometer.

The clinical thermometer is so indispensably part and parcel of our diagnostic armament that it is hard to realise how comparatively modern its invention is, and how grudgingly it was at first adopted by the profession. Yet the intricacies of the thermics of the human body in health and disease are such that no one would be bold enough to deny our profound ignorance of much that ought to be, and some day will be, made plain. Not very long ago attention was drawn to the studies of temperature in normal healthy women. Those researches, incomplete as they were, yet indicated how much work is still to be done in this field. As an instrument of precision the modern clinical thermometer is well enough; but it would seem, from an announcement in the daily Press, that a much more delicate apparatus has been perfected by a South Kensington scientist, Professor Callendar, which not only registers the human temperature with great exactitude, but also keeps automatically a continuous chart showing every fluctuation for as long a period as the machine is bandaged into the axilla. From the description of this ingenious and refined piece of mechanism it would seem doubtful whether it is ever likely to be of much service to the general practitioner; but experience has taught physicians never to despise, always to welcome, any apparatus which makes for greater accuracy in estimating physical signs. Whether the continuous observation of temperature thus rendered possible will turn out of any practical value, future researches must decide; if it does, the co-operation of the physicist with the physician may in time be extended to other fields of clinical medicine.

